# Total antioxidant capacity – a novel early bio-chemical marker of oxidative stress in HIV infected individuals

**DOI:** 10.1186/1423-0127-16-61

**Published:** 2009-07-07

**Authors:** DR Suresh, Vamseedhar Annam, K Pratibha, BV Maruti Prasad

**Affiliations:** 1 Department of Biochemistry, Sri Siddhartha Medical College, Tumkur, India; 2 Department of Pathology, Sri Siddhartha Medical College, Tumkur, India; 3 Department of Biochemistry, Bangalore Medical College, Bangalore, India

## Abstract

**Background:**

Oxidative stress induced by the production of reactive oxygen species may play a critical role in the stimulation of HIV replication and the development of immunodeficiency. This study was conducted as there are limited and inconclusive studies on the significance of a novel early marker of oxidative stress which can reflect the total antioxidant capacity in HIV patients,

**Methods:**

Total antioxidant capacity (TAC) and lipid peroxidation were evaluated in 50 HIV-1 seropositive patients (including HIV-1 symptomatics and asymptomatics). Controls included 50 age and sex matched and apparently healthy HIV-1 seronegative subjects. Serum malondialdehyde (MDA), Total antioxidant capacity [TAC] (by ferric reducing antioxidant power assay), vitamin E, vitamin C and superoxide dismutase (SOD) enzyme activity were estimated among controls and cases. Statistical comparisons and correlations at 5% level of significance were determined.

**Results and Discussion:**

The mean MDA concentrations were significantly elevated in both HIV-1 asymptomatic (CD4+ count > 500 cells/microliter) and HIV-1 symptomatic (CD4+ count <500 cells/microliter) groups (Mean ± S.D values were 2.2 +/- 0.7 nmol/ml and 2.8 +/- 0.8 nmol/ml respectively) when compared with the control group (Mean ± S.D value was 0.9 +/- 0.2 nmol/ml) (p < 0.01). The mean TAC of HIV- 1 asymptomatic and HIV-1 symptomatic (Mean ± S.D values were 754.6 ± 135.6 μmol/L and 676.6 ± 154.1 μmol/L respectively) patients were significantly reduced compared with the control group (Mean ± S.D value was 1018.7 ± 125.6 μmol/L) (p < 0.01). Also, there were significantly decreased levels of vitamin E, vitamin C and SOD among HIV-1 seropositive patients(controls > asymptomatic > symptomatic) compared to controls (p < 0.01). TAC showed significant negative correlation with MDA among HIV-1 infected patients (p < 0.01).

**Conclusion:**

Our results clearly show that severe oxidative stress occurs in the HIV-1 seropositive patients in comparison with controls, and increases significantly with the progression of disease, i.e. HIV-1 symptomatics > asymptomatics > controls. TAC can be used as a novel early bio-chemical marker of oxidative stress in HIV-1 infected patients which may result in reduced tissue damage by free radicals and help to monitor and optimize antioxidant therapy in such patients.

## Background

HIV infection is characterized by numerical and functional decline in CD4 cells, which result in progressive immunodeficiency. Oxidative stress can be defined as an imbalance between the oxidant and antioxidant system, with an advantage towards the oxidant system: A variety of enzymatic (superoxide dismutase, catalase, glutathione peroxidase etc.) and nonenzymatic (carotenoids, tocopherols, ascorbate, bioflavonoids, bilirubin, uric acid etc) antioxidants present in human serum become insufficient to circumvent HIV-1 replication secondary to cellular ROS production (superoxide anion, hydroxyl radical, hydrogen peroxide etc) by a pro-oxidant effect of inflammatory cytokines and/or a polymorphonuclear leukocyte activation [1]. No single component of serum antioxidant complex could fully reflect the protective efficiency of blood, probably because of interactions that occur in vivo among different antioxidant compounds. Total antioxidant capacity (TAC) considers the cumulative effect of all antioxidants present in blood and body fluids [2]. Although previous studies have suggested a role of oxidative stress in the stimulation of HIV replication and the development of immunodeficiency, significance of a novel early marker of oxidative stress which can reflect the total antioxidant capacity in HIV patients is not much studied [1,2].

The purpose of the present study was to measure and compare the TAC and lipid peroxidation in HIV-1 asymptomatic and HIV-1 symptomatic patients with age matched HIV-seronegative healthy controls and to correlate the lipid peroxidation with antioxidant markers.

## Materials and methods

This study was conducted at Sri Siddhartha Medical Teaching Hospital and Research Centre, Tumkur. The study included 50 cases aged >18 years, of either sex (31 men with mean age of 45.4 years and 19 women with mean age of 36.2 years), diagnosed as HIV-1 infected patients (confirmed by TriDot ELISA) before the commencement of antiretroviral therapy and 50 age and sex matched healthy controls. HIV-1 seropositive patients were further classified according to the Centre for Disease Control and Prevention (CDC) Criteria as HIV-1 asymptomatic (CD4+ count > 500 cells/microliter) [n = 28] and HIV-1 symptomatic including AIDS patients (CD4+ count < 500 cells/microliter) [n = 22]. All subjects underwent an initial screening that included a detailed history (medical, smoking, diet, and alcohol and supplemental vitamin intakes) and anthropometric (weight and height) and biochemical (complete blood count, glucose, creatinine, urea, and liver enzymes) measurements. Patients were eligible if they had no acute opportunistic infection. Exclusion criteria were as follows: smoking, patients on antioxidant vitamin therapy before the study, hyperlipidemia, diabetes, kidney or liver dysfunction, intractable diarrhea (more than six liquid stools per day), vomiting, or evidence of gastrointestinal bleeding. The study was conducted after informed consent was obtained and the study has been approved by the ethical committee of Sri Siddhartha Medical College, Tumkur.

Under aseptic precautions about 6 ml of a venous blood sample was collected from HIV-1 patients and from healthy controls. Blood was collected in appropriate tubes and centrifuged at 3000 g for 15 min and the separated serum was stored at 4°C until analysis was carried out. Ferric reducing antioxidant power (FRAP) assay, estimation of malondialdehyde (MDA), vitamin E, vitamin C levels and SOD enzyme activity were carried out on serum sample. All the chemicals used were of highest analytical grade available in India. CD4+ count, CD8+ count and CD4+/CD8+ ratio were measured by Tricolor flowcytometry.

Total antioxidant capacity was measured by FRAP assay according to the method of Benzie.F.F. and J.J.Strain [3]. At low pH, when a ferric tripyridyltriazine (Fe ^III^-TPTZ) complex is reduced to the ferrous (Fe ^II^) form, an intense blue colour with an absorption maximum at 593 nm develops.

Serum vitamin E was estimated by Baker and Frank method [4] and vitamin C by 2, 4 – DNPH method [5]. SOD enzyme activity was determined with the direct spectrophotometric method employing KO2 as previously described by Marklund, with some modifications [6,7]. The data are presented as units per milliliter of serum.

Lipid peroxidation was measured by serum MDA estimation according to the colorimetric method of Satoh.k [8]. Lipoproteins are precipitated from the specimen by adding trichloroacetic acid. 0.05 M sulphuric acid and 0.67% thiobarbituric acid (TBA) in 2 M sodium sulphate are added to this precipitate and the coupling of lipid peroxide with TBA is carried out by heating in a boiling waterbath for 30 minutes. The resulting chromogen is extracted in n-butanol, which is measured colorimetrically at 530 nm.

### Statistical analysis

Each result was expressed as mean ± standard deviation. The statistical significance of the data were determined by Student's t-test and one way ANOVA test at 5% level of significance. Pearson's correlation coefficient was determined at 5% level of significance. Statistical analysis was done using SPSS software version 16.0.

## Results

There was significantly increased levels of MDA in the HIV-1 seropositive patients (HIV-1 symptomatic > HIV-1 asymptomatic) compared to the control group (p < 0.01) (Table 1) suggesting lipid peroxidation. There were significantly decreased levels of vitamin E, vitamin C, SOD enzyme activity along with TAC in the HIV-1 seropositive patients (Significantly decreased in HIV-1 symptomatics compared to HIV-1 asymptomatics) compared to the control group (p < 0.01) (Table 1), suggesting antioxidant depletion. Statistically significant negative correlations found between TAC and MDA in both HIV-1 symptomatic and HIV-1 asymptomatic patients suggested oxidative stress (Table 2). Our results clearly show that severe oxidative stress occurs in the HIV-1 seropositive patients in comparison with controls, and increases significantly with the progression of disease, i.e. HIV-1 symptomatics > asymptomatics > controls.

**Table 1:** Comparisons of oxidants and antioxidants in HIV-1 infected patients.

	**Controls (MEAN ± S.D) N = 50**	**HIV-1 Asymptomatic patients (MEAN ± S.D) N = 28**	**HIV-1 Symptomatic patients (MEAN ± S.D) N = 22**	**p value**
**MDA (n mol/mL)**	0.9 ± 0.2	2.2 ± 0.7	2.8 ± 0.8	P < 0.01

**TAC (μmol/L)**	1018.7 ± 125.6	754.6 ± 135.6	676.6 ± 154.1	P < 0.01

**Vitamin E (mg/L)**	10.0 ± 0.8	8.2 ± 0.6	7.4 ± 0.6	P < 0.01

**Vitamin C (mg/dl)**	6.3 ± 0.2	5.8 ± 0.2	3.6 ± 0.2	P < 0.01

**SOD activity (units/ml)**	8.75 ± 1.1	4.9 ± 0.4	3.7 ± 0.4	P < 0.01

MDA – Malondialdehyde, TAC – Total Antioxidant Capacity, SOD – Superoxide Dismutase.

**Table 2:** Correlations between oxidants and antioxidants in HIV infected patients.

	**HIV-1 Asymptomatic patients**		**HIV-1 Symptomatic patients**	
	**r value**	**p value**	**r value**	**p value**

**MDA & TAC**	- 0.56	< 0.01	- 0.74	< 0.01

## Discussion

Infection by human immunodeficiency virus (HIV) causes persistent chronic inflammation. Viral Tat protein plays a role in the intracellular increase of reactive oxygen species which in turn influence the increase in the apoptosis index, mainly the one mediated by CD95 causing a decrease of CD4 + T cell lymphocytes and more importantly an increase in HIV-1 replication secondary to free radicals overproduction [9]. Under normal conditions, the ROS produced in the course of metabolism are contained by the natural antioxidant system which consists of a series of antioxidant enzymes such as superoxide dismutase (SOD), catalase, glutathione peroxidase as well as numerous endogenous and dietary antioxidant compounds that are capable of reacting with and inactivating ROS thereby protects the functional and structural molecules against ROS-mediated tissue damage [10].

MDA is a three carbon, low molecular weight aldehyde that can be produced from free radical attack on polyunsaturated fatty acids of biological membranes. The determination of MDA is used for monitoring lipid peroxidation in biological samples. Previous studies show inconsistent findings regarding MDA levels in HIV-1 infected patients (lipid peroxidation is similar between HIV asymptomatics and HIV symptomatics) [11]. In the present study, the significantly elevated serum MDA concentration in HIV-1 infected patients (HIV-1 symptomatics > HIV-1 asymptomatics, in contrast to previous studies) compared to controls correlating negatively with TAC reflects the increased formation of ROS and lipid peroxidation with progressive immunodeficiency.

Vitamin E, a potent chain breaking lipid soluble antioxidant, reacts with lipid peroxyl radicals eventually terminating the peroxidation chain reaction and thereby reducing oxidative damage. Vitamin C represents the major water-soluble antioxidant in the human body. SOD is an endogenous antioxidant that catalyses the dismutation of the superoxide anion radical [12]. There are conflicting reports in the values of antioxidant vitamins (vitamin E and vitamin C) and SOD enzyme activity among HIV-1 infected patients in various stages in the literature [11,12]. In our study, vitamin E, vitamin C, SOD and TAC levels are decreased in HIV-1 patients (depletion is pronounced in HIV-1 symptomatics compared to HIV-1 asymptomatics, in contrast to previous studies where there were no significant differences in antioxidant vitamins in both groups [11]) which is observed from HIV-1 asymptomatic stage itself.

Although the concentration of plasma antioxidant components can be measured individually, these measurements may be time- and cost-consuming and labour intensive. In addition, it may not accurately reflect the total antioxidant status [13]. Thus, the accurate antioxidant capacity of the organism can only be determined by the measurement of total antioxidant capacity. FRAP assay is presented as a novel method of assessing total antioxidant capacity and is considered as a useful indicator of the body's antioxidant status to counteract the oxidative damage due to ROS. The advantage of the FRAP assay is in being fast, easy to handle, with highly reproducible results [14]. A significantly lower serum TAC concentration in the HIV-1 patients compared to controls reflects a lower total antioxidant capacity. Negative correlation between MDA and TAC in this study suggests an increased oxidative stress in HIV-1 infected patients. This antioxidant deficiency in HIV-1 seropositive populations is probably due to depletion of antioxidant molecules when they are consumed in the process of protecting cells against ROS induced oxidative damage in a magnitude that is related to advancement of the disease to AIDS. A weakened antioxidant defense system, in turn, could lead to further enhancement in lipid peroxidation [3,15].

In conclusion, this study showed increased lipid peroxidation, measured by MDA concentrations, in HIV-1 seropositive patients and decreased concentrations of individual antioxidants and total antioxidant capacity which was evident from HIV-1 asymptomatic stage itself. The possibility of counteracting oxidative stress by a pool of proper antioxidants plus an appropriate diet, mainly in patients whose blood antioxidant deficiencies can be easily rebalanced, may have real health benefit and represent a promising way of inhibiting the progression of disease. Thus, TAC may be useful as an early marker of oxidative stress to monitor and optimize antioxidant therapy as an adjunct in the management of HIV-1 infected patients.

## Competing interests

The authors declare that they have no competing interests.

## Authors' contributions

DRS planned the design of the study, carried out the biochemical analysis and drafted the manuscript. VA participated in the design of the study and carried out CD4+, CD8+ counts. KP participated in the design of the study and performed the statistical analysis. BVMP participated in its design and coordination. All authors read and approved the final manuscript.
